# High detectability costs select weak warning signals in chemically defended gregarious prey

**DOI:** 10.1093/beheco/arag010

**Published:** 2026-02-02

**Authors:** Katja Koskenpato, Timo Mikkonen, Johanna Mappes, Janne Valkonen, Carita Lindstedt

**Affiliations:** Department of Forest Sciences, Faculty of Agriculture and Forestry, University of Helsinki, Latokartanonkaari 7, Helsinki 00790, Finland; Department of Biological and Environmental Science, University of Jyväskylä, Seminaarinkatu 15, Jyväskylä 40100, Finland; Organismal and Evolutionary Biology Research Programme, Faculty of Biological and Environmental Sciences, University of Helsinki, Viikinkaari 1, Helsinki 00790, Finland; Department of Biological and Environmental Science, University of Jyväskylä, Seminaarinkatu 15, Jyväskylä 40100, Finland; Department of Forest Sciences, Faculty of Agriculture and Forestry, University of Helsinki, Latokartanonkaari 7, Helsinki 00790, Finland

**Keywords:** warning coloration, antipredator behavior, crypsis, social behavior, insects, predator–prey interactions

## Abstract

The evolution of costly chemical defenses in cryptic prey is puzzling, as conspicuousness should be adaptive for defended prey by enhancing predator avoidance learning. Here, we explore three hypotheses that may promote weak visual signal strategies: (1) Low prey conspicuousness is favored in diverse predator communities; (2) Conspicuousness is less critical if color patterns or behavior allow defended prey to be distinguished from cryptic prey; and (3) in gregarious prey species, aggregation itself could act as a deterrent signal. We used chemically defended and gregarious European pine sawfly (*Neodiprion sertifer*) larvae, exhibiting green-black-grey coloration, as our study system. First, we manipulated the conspicuousness of the larvae by placing them either solitarily or in groups and marking them with either cryptic or conspicuous coloration, then testing their survival against predators in the field. Second, we analyzed how conspicuous the ventral side (which larvae reveal during a defense display) of *N. sertifer* appears to avian predators and how its conspicuousness correlates with the larvae's chemical defenses. Our results indicate that increased conspicuousness was costly and decreased larvae survival. The ventral side of larvae making the defense display was more conspicuous (showed higher luminance) than that of larvae not displaying; however, this was not associated with chemical defense traits. The signaling function of synchronous displays involving defensive secretions in sawflies presents a promising avenue for future studies on the mechanisms by which weak visual signals operate in nature.

## Teaser

Why do some chemically defended prey use weak visual signals when conspicuous aposematic coloration seems more effective for predator avoidance? Using weakly conspicuous pine sawfly larvae as a study system, we found that increased conspicuousness under predation risk from natural predator communities reduces their survival. Our findings highlight the importance of understanding ecological mechanisms behind the evolution of weak visual signal strategies.

## Introduction

Animals use visual, acoustic, and chemical signals to convey defense status, social dominance, or mate quality. Yellow-black color patterns of bees warning predators about the painful sting, or male cricket's calling song for attracting conspecific females (Zuk et al. 2006), are just a few examples of different signaling systems in the animal kingdom. A signal is defined as any trait or act that alters the behavior of a receiver, evolved due to its effect, and is maintained by the receiver's evolved response (Ruxton et al. 2004). Often conflict arises because even though the detectability of the signals ensures that the message is properly delivered from signaler to receiver, conspicuousness of the signal may invite attacks from enemies such as predators or parasites (Maynard Smith and Harper 2003; Endler and Mappes 2004; Zuk et al. 2006; Lindstedt et al. 2008; de Roode and Groot 2025). Signals can also be expensive to produce and trade off with other important fitness traits, depending on the individual's condition and environment (Maynard Smith and Harper 2003; Zuk et al. 2006; Speed and Ruxton 2007; Lindstedt et al. 2009, 2010). As a result of these different selection pressures on signal expression, signaling strategies have evolved to be extremely diverse in the animal kingdom.

Variation in defensive coloration and how it co-evolves with secondary defenses such as spines or toxicity provides a good opportunity to test the conditions under which signals evolve. Protective coloration in animals forms a continuum, where at one extreme color patterns are based on the prevention of detection by predators such as camouflage (nonsignaling coloration), and at the other extreme highly detectable color patterns (signaling coloration) inform predators that the individual is toxic, unpalatable, or unprofitable (ie aposematism) (Ruxton et al. 2004; Mappes et al. 2005). The benefit of a highly detectable warning signal is that it ensures that the prey is recognized quickly by a predator which has previous bad experience of it, and an attack stops before the prey is lethally injured (Gamberale-Stille and Tullberg 1999; Maynard Smith and Harper 2003; Ruxton et al. 2004). Thus, it is in the mutual interest of both the prey and the predator to offer effective cues or signals of unprofitability to a predator, decreasing the attack risk (Gamberale-Stille and Tullberg 1999; Lindström et al. 1999; Ruxton et al. 2004; Lindstedt et al. 2008).

To date, research has mainly concentrated on understanding the form and function of these two extremes (Ruxton et al. 2004). However, between these extremes are many species that are inconspicuously colored but still possessing chemical defenses (Endler and Mappes 2004; Loeffler-Henry et al. 2023) such as the moth *Utetheisa galapagensis* (Lepidoptera, Arctiidae) (Roque-Albelo et al. 2002), larvae of many pine sawflies (Endler and Mappes 2004; Lindstedt et al. 2006; 2011) or shield bugs (Chinery 1993). As conspicuous signals should enhance the predator's avoidance learning efficiency (Gamberale-Stille and Tullberg 1999; Lindström et al. 1999; Lindstedt et al. 2008), the occurrence of these secondary defended prey species with low signal intensity is puzzling; especially if chemical defenses are costly to produce and maintain (Endler and Mappes 2004) and an individual is already well protected by camouflage.

At present, it is assumed that weak visual warning signals (defined here as color patterns that do not include any typical aposematic colors such as red, orange, yellow and white combined with black elements) can evolve if the predators vary in their tendency to attack defended prey and may cause high predation pressure for conspicuous prey (Endler and Mappes 2004). For example, different species of passerines differ in their tendency to attack a conspicuous firebug (*Pyrrhocoris apterus*) suggesting that warning coloration does not have a universal antipredatory effect (Exnerová et al. 2003). In addition to the spatial structure of a predator community, its temporal fluctuation and age structure also affect the detectability risk (Mappes et al. 2005, 2014). For example, conspicuous warning signals improve survival when naïve young bird fledglings are rare, but the reverse is true when the number of fledglings peak (Mappes et al. 2014). Predation risk of aposematic prey can also be affected by the number of alternative prey available to predators (Merilaita and Kaitala 2002; Lindström et al. 2004). If high-quality prey is scarce, predators are more likely to consume low-quality ie defended prey (Richards 1983; Kokko et al. 2003; Beatty et al. 2004; Elkinton et al. 2004; Sandre et al. 2010). Conspicuousness may also increase the risk of being detected by parasites (Zuk and Kolluru 1998; Zuk et al. 2006). If the costs of conspicuousness exceed the benefits, it is not worth allocating resources to conspicuous coloration. Thus, prey may gain the best fitness by being intermediately or weakly conspicuous (Endler and Mappes 2004; Ruxton et al. 2009). It is also possible that conspicuousness may not be as important if the signal is distinguishable from the cryptic prey (Ruxton et al. 2004) based on eg distinctive pattern, smell or behavior (Merilaita and Ruxton 2007). Conspicuous coloration may also be unnecessary for defended prey if the predator releases the prey soon after attacking and the prey survives (Wiklund and Järvi 1982). Finally, if the species is gregarious, the high-density itself could act as a signal (Alatalo and Mappes 1996; Riipi et al. 2001; Beatty et al. 2004). For example, Riipi et al. (2001) showed that unpalatable prey survived better in groups regardless of coloration as predators learn to associate a group to unpalatability and thus per capita mortality in groups was reduced. Group living can also be beneficial simply via dilution effect (ie predation risk per se decreases in groups) (Sillén-Tullberg 1990; Riipi et al. 2001).

**Figure 1:**
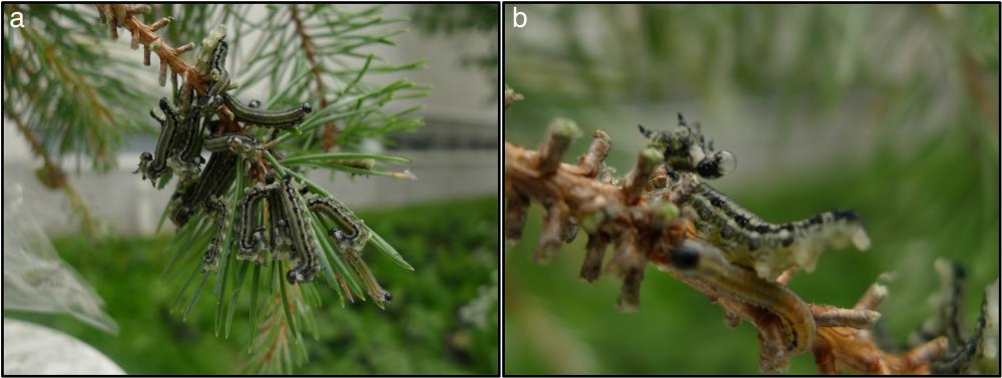
A group of defending *N. sertifer* larvae a). A larva exposing its ventral side and producing defense fluid when threatened b). Photos: Carita Lindstedt.

To determine the conditions that select for weak visual signal strategies, we used the European pine sawfly (*Neodiprion sertifer*) larvae as a study system (Lindstedt et al. 2025). *N. sertifer* larvae are chemically defended against avian (Sillén-Tullberg 1990; Lindstedt et al. 2011) and invertebrate predators (Eisner et al. 1974; Lindstedt et al. 2006). Larvae appear weakly conspicuous to human eyes (Lindstedt et al. 2011) and live in dense aggregations throughout their entire larval stage until the prepupal instar. Group living in pine sawfly larvae serves multiple functions. For example, group living facilitates feeding on pine needles, especially in early larval instars (Ghent 1960). Additionally, gregarious pine sawfly larvae perform a group defense, divided into two stages. When threatened by a predator, the larvae raise their heads and tails (ie U-posture) synchronously as a first line of defense. The defensive U-posture is also required for them to proceed to the second stage of the collective defense, where the larvae deploy the resinous sticky fluid from their mouths (Björkman and Larsson 1991; Codella and Raffa 1995; Lindstedt et al. 2018) (Fig. 1). The key compounds in the defensive fluid are monoterpenes and diterpenes, which the larvae sequester from their diet (*Pinus sylvestris*) and store in separate defensive pouches (Eisner et al. 1974; Björkman and Larsson 1991; Codella and Raffa 1995; Lindstedt et al. 2006, 2011). Higher resin content of the host plant increases the efficacy of larval defense (Larsson et al. 1986; Björkman and Larsson 1991; Codella and Raffa 1995; Lindstedt et al. 2006). However, high resin content of host plant is also costly to the larvae and decreases larval performance in terms of growth (Björkman et al. 1991; Björkman and Larsson 1991; Lindstedt et al. 2018).

Deploying the fluid is a facultative trait that can be considered a collective good (ie larvae producing the fluid aid also the survival of their group members) (Lindstedt et al. 2018; Gupta et al. 2025; Ritter et al. 2025; Van Meyel et al. 2025). The production of fluid is costly for the pine sawfly larvae, decreasing their growth, immune defense, and future defensive capacity against predators (Björkman and Larsson 1991; Lindstedt et al. 2018). An individual's likelihood of deploying the fluid varies among individuals; it is female-biased and varies among larval colonies (Lindstedt et al. 2018, 2025; Ritter et al. 2025). However, selection by predators should promote fluid deployment, as individuals have a higher likelihood of surviving predation when they are in a defensive group (Ritter et al. 2025; Van Meyel et al. 2025). Deploying the fluid also provides individual benefits for the larvae by directly improving their survival (Eisner et al. 1974; Codella and Raffa 1996; Ritter et al. 2025). The proportion of individuals displaying the fluid depends on the social environment (Ritter et al. 2025; Van Meyel et al. 2025). There are always more individuals participating in the less-costly first stage of the group defense (U-posture), and the proportion of individuals participating increases with group size (Van Meyel et al. 2025). However, the proportion of individuals deploying the fluid (ie the more costly stage of the defense) decreases as group size increases (Van Meyel et al. 2025; see also Daly et al. 2012).

In addition to its collective defense behavior (Lindstedt et al. 2025), pine sawflies provide a representative taxonomic group for studying the evolution of warning coloration (Linnen et al. 2018). Protective color strategies in group-living pine sawflies vary from nearly cryptic to traditional aposematic white-to-black and yellow-to-black pigmentation (Linnen et al. 2018; Lindstedt et al. 2022). In some pine sawfly species, such as *Neodiprion lecontei*, coloration and its conspicuousness show extensive local variation (Linnen et al. 2018; Lindstedt et al. 2025). Based on previous work on aposematically colored *N. lecontei* pine sawflies, the more conspicuous aposematic pigmentation should improve survival against predators by enhancing avoidance (Lindstedt et al. 2022). Yet, many gregarious and chemically defended pine sawfly species display cryptically or weakly conspicuous (eg black patterns combined with green or yellowish-green background) color patterns. This variation provides a good opportunity to study its sources.

We had two main aims in this study. Our first aim was to determine the potential costs of being conspicuous in a natural environment, in which the visually hunting predator community is expected to be diverse, and the predators will vary in their experience, age and prey preferences (Marples et al. 1998; Exnerová et al. 2003; Thomas et al. 2004; Marples et al. 2005; Medina et al. 2025). To study this, we conducted a field experiment where we manipulated coloration and group size of *N. sertifer* larvae. We hypothesized that natural selection by predators may favor less conspicuous coloration in a chemically defended species if the efficacy of the chemical defense varies among predators (Endler and Mappes 2004). Under this scenario, increasing conspicuousness (by manipulating color and/or grouping) will increase predation risk, as predators that are less susceptible to the chemical defenses of *N. sertifer* larvae are more likely to detect them. Alternatively, conspicuous coloration may enhance survival of the larvae if it improves the avoidance learning efficacy of predators (Sillén-Tullberg 1985, 1990; Alatalo and Mappes 1996; Mappes and Alatalo 1997; Lindström et al. 1999; Riipi et al. 2001). This can be further enhanced in groups of prey by amplifying predator learning (Greenwood et al. 1989; Endler 1991; Ruxton et al. 2004).

Our second aim was to explore the potential signal function of the defensive display of larval groups. More specifically, we measured how revealing the ventral side of the larvae during the defensive display (hereafter referred to as “U-posture”, Fig. 1b) may affect their conspicuousness to predators and how the brightness of ventral side correlates with the larval chemical defense behavior. The visual systems of humans and birds differ, as only birds can sense the UV spectrum of light due to different numbers of cone types in bird and human retina (Jacobs 1992; Cuthill 2006). For this reason, we used an avian vision model (Vorobyev and Osorio 1998; Vorobyev et al. 1998) to investigate how a potential predator, a blue tit (*Cyanistes careuleus*), perceives the coloration of the ventral side of a *N. sertifer* larva. Furthermore, if conspicuousness of the ventral coloration would act as an honest signal of chemical defense in *N. sertifer* larvae, we expected to see a positive correlation between conspicuousness (see Conspicuousness of the coloration of *N. sertifer* larvae to avian predators in Materials and methods) and an individual's defensive display (U-posture), as well as potency of the individual's chemical defense (ie whether the individual does the responsive defense and how much fluid it produces).

## Materials and methods

### *N. sertifer* insect cultures

*N. sertifer* is common in North European pine forests where larvae are specialized in feeding on needles of Scots pine (*Pinus sylvestris*). The species have regular outbreaks (Larsson and Tenow 1984) and defoliation during the outbreaks decreases tree growth and causes economic losses (Larsson et al. 1993). In Finland, females lay overwintering eggs in needles during late summer. Larvae hatch early in the next summer, starting immediately to feed in groups (Kolomiets et al. 1979; Davis et al. 2023; Lindstedt et al. 2025).

To acquire the experimental individuals for the predation experiment, we collected pine branches with *N. sertifer* eggs from a former outbreak area in Puumala, Finland (61°31′25″N, 28°10′40″E) in 2013 and brought them into a laboratory for rearing. The rearing was conducted under standardized conditions with constant room temperature of +18 °C. After hatching, the larvae (P-generation) were provided with fresh pine branches ad libitum, at least twice a week. Part of the larval groups from P-generation were used for the predation experiment (see below), while other groups were reared until adulthood. After pupation and following eclosion, adults were mated, avoiding inbreeding by pairing only individuals originating from different branches as eggs (individuals from the same branch were assumed to be siblings and are hereafter referred to as “family”) (Terbot and Linnen 2019; Ritter et al. 2025). Mating pairs were taken to the field on pine branches covered with mesh bags to prevent the adults from flying away. Using living pine trees as breeding grounds is necessary, as *N. sertifer* overwinters in the egg stage, and the eggs require a long cold period to break diapause (Heliövaara et al. 1990; Björkman et al. 1991). The eggs remain viable only inside the living needles. The following spring 2014 the mating branches were cut off from the tree and taken inside the laboratory to enable overwintered eggs to hatch. Branches were kept in water and the tips of the branches were cut every second day to maintain water uptake. Hatched larvae (F1-generation) were reared similarly to the P-generation described above.

### The effect of increased conspicuousness and gregariousness on predation risk under a natural predator community

In the field predation experiment, larval conspicuousness was manipulated by painting either a yellow (conspicuous) or a green (cryptic) spot (diameter ∼1.5 mm) with water soluble nontoxic acrylic paints (FolkArt) on the dorsal side of the larva. We collected a preliminary data showing that neither of the paints used affected *N. sertifer* larval mortality (see Supplementary material for further description).

The predation risk of larvae under different color and group size treatments was studied in the field. To manipulate gregariousness, larvae from the P-generation were divided into solitary and group treatments (ten larvae per group) following a split-family design. Therefore, each pair of solitary and group treatment larvae originated from the same wild collected larval colony. We kept larvae from the same family together to control potential genetic variation in larval coloration and responsive defense behavior. In total, larvae from 50 families were included in the experiment to represent a reliable sample of natural variation in coloration and defense between families. To manipulate larval conspicuousness, we used yellow paint because it is one of the most widely occurring colors related to warning coloration in nature (Wallace 1889; Sherratt and Beatty 2003). Green paint was used as a control for potential paint smell or other effects with no intention to alter larval natural appearance regarding coloration. We used an avian vision model to verify how the paint treatment impacted the conspicuousness of the larvae. We measured dorsal reflectance from three larvae with green paint and from three larvae with yellow paint. We took measurements from both the unpainted and painted parts of the dorsal side to compare the detectability of the painted color patch against the natural larval pigmentation. We then used the mean cone catch values of these measurements for the just noticeable difference (JND) comparison (see Conspicuousness of the coloration of *N. sertifer* larvae to avian predators in the Materials and methods for more details on the avian vision model and JND values). Yellow paint on the dorsal side increased the conspicuousness of the larvae, with a color contrast JND of 20.2 and a luminance contrast JND of 29.5. In contrast, the green paint did not change the conspicuousness relative to the natural coloration (for the paint against the dorsal side of the larvae: color contrast JND = 1.6 and luminance contrast JND = 5.8), which were lower values than those of pine needles against the dorsal side of the larvae (1.8 and 7.8, respectively). A control treatment, where predators were excluded, controlled mortality caused by other potential factors than predation, eg nuclear polyhedrosis virus which is a common pathogen in *N. sertifer* pine sawflies in wild (Bird and Whalen 1953). This was done by covering a branch with a mesh bag to prevent predation. In general, pine sawfly larvae do not drop from branches as a predator avoidance mechanism and do not leave the branch as long as they have food available (Lindstedt et al. 2006).

All experimental larvae were weighed before dividing into treatments to ensure that the size of the larvae did not differ significantly among treatments (*P* = 0.56 for solitary larvae and *P* = 0.91 for larvae in groups, see Supplementary material for more detailed description). In total, the experiment consisted of eight different treatments: (1) a conspicuous solitary larva (20 replicates), (2) group of 10 conspicuous larvae (20 replicates), (3) a cryptic solitary larva (22 replicates), (4) group of 10 cryptic larvae (19 replicates), (5) a conspicuous solitary larva in a mesh bag (9 replicates), (6) group of 10 conspicuous larvae in a mesh bag (10 replicates), (7) a cryptic solitary larva in a mesh bag (10 replicates) and (8) group of 10 solitary larvae in a mesh bag (9 replicates).

The experiment was conducted at a study site in Leppälahti, Finland (62°14′93″N, 26°02′20″E) in June 2013. The area consisted of 10- to 20-yr-old pure pine stands and mixed forests suitable for *N. sertifer*. Thus, while study area is typical habitat for *N. sertifer*, it is not abundant in the area and the predator community structure and abundance reflects the conditions prevailing in the low population density-stage.

Two transects were established in the study area. On each transect, larvae were placed on experimental branches ∼150 cm off the ground, with one treatment per experimental branch per tree. To prevent ant predation on the larvae, square-shaped barriers made of thin transparent plastic plates covered with liquid silicon were attached around the experimental branches (Lindstedt et al. 2006). The barriers also prevented the larvae from moving away from the branches. The distance between pine trees with experimental branches was a minimum of 10 m to minimize the chance that a predator would encounter multiple treatments. The order of the treatments within each transect was randomized, and this same order was repeated throughout both transects. The experiment lasted for five consecutive days, during which all larval branches were checked daily to document larval mortality and replace dead larvae with new ones to maintain the odds ratio of group size treatments. All missing larvae were assumed dead and eaten by a predator. For all the mesh bag treatments, however, dead larvae were only counted at the end of the experiment to avoid the disturbance caused by the daily opening and closing of the mesh bags. Weather during the experiment was typical for a Finnish summer, with some warm sunny days and some colder rainy days.

### Conspicuousness of the coloration of *N. sertifer* larvae to avian predators

We used a common-garden design with 13 full-sib families from the F1-generation to measure different color and chemical defense traits. Ten days after hatching, 10 larvae from each family were randomly chosen for further rearing under standardized lab conditions (constant temperature 20 °C and natural light coming from the windows together with some electric light). The larvae were kept in plastic containers with fabric covers on top for ventilation. Group sizes were standardized to 10 larvae to avoid any potential effects of group size on larval traits. Since resin acid concentration may vary among pine trees and affect the chemical defense of the larvae (Eisner et al. 1974; Björkman and Larsson 1991; Lindstedt et al. 2006), all reared larvae were provided with a similar mixture of different pine branches for food at least twice a week. Larval boxes were sprayed daily with water.

To analyze the conspicuousness of different stimuli to avian predators, we first measured the reflectance of these different objects. For each larva, we measured reflectance from both the ventral and dorsal sides with a spectrophotometer (Ocean Optics USB4000) at intervals of 0.47 nm across a wavelength range of 300 to 750 nm. From the three values obtained per nanometer, only the first one was used in the subsequent analyzes (Nokelainen et al. 2012). As a background, we used pine needles collected from six randomly chosen pine trees growing at the same site where the larval food branches were gathered. The distance between trees was 100 to 500 m. The reflectance of the needles from each tree was measured separately and their mean reflectance was used in the vision model analyzes. We also measured the reflectance of the defensive fluid from a pooled sample collected from several individuals. All reflectance measurements were conducted by placing the object in a petri dish placed on black velvet to minimize reflection from the dish.

We then used MATLAB to conduct avian vision models (Vorobyev and Osorio 1998; Vorobyev et al. 1998) to calculate color contrasts between the larval color pattern elements, the background (mean reflectance of needles) and the defensive fluid (similarly to Nokelainen et al. 2012; Lindstedt et al. 2022). We studied the variation between individuals in color and luminance contrasts of dorsal and ventral sides against pine needles. Contrasts between defense fluid against the dorsal and ventral side of the larva itself, and against pine needles, were also measured. The avian vision model considers specific characteristics of blue tit vision, eg sensitivity of different receptors and their relative abundance, enabling the assessment of how well blue tits can discriminate the objects of interest (Vorobyev et al. 1998).

The avian vision model uses information on blue tit vision in midday luminal conditions (D_65_). Birds' ability to discriminate hues is based on four types of single cones (Cuthill 2006), whereas the ability to sense brightness is based on double cones (Osorio and Vorobyev 2005). This information is integrated in the avian vision model by using a Weber fraction of 0.05 for the most abundant cone type (Siddiqi et al. 2004). The avian vision model uses units called just noticeable differences (JNDs) when comparing color (hues) or luminance (brightness) contrasts (Siddiqi et al. 2004). Values <1 indicate that the two objects are likely indistinguishable, values 1 to 3 indicate that the two objects are likely distinguishable but only under optimal light conditions, and values >3 indicate that two objects are likely distinguishable. We note that these simulations only estimate the conspicuousness of color on the measured stimulus and do not take into account the effect of color pattern on the conspicuousness of pine sawfly larvae for blue tits.

### Phenotypic correlation between *N. sertifer* larval coloration and chemical defense

During their last instar prior to the prepupal stage, five larvae from each family were randomly chosen for phenotypic measurements. We measured (1) the length of the larva, (2) defensive movement (U-posture/no U-posture), (3) production of defense fluid (yes/no), (4) the volume of produced defense fluid, and (5) the conspicuousness of the larva and its defense fluid to a bird predator.

To assess defensive behavior, each larva was gently poked once with a capillary tube on the dorsal side and the presence of the U-posture (yes/no) and the production of defense fluid (yes/no) was recorded. The produced defense fluid was collected into a capillary tube, and the length of the regurgitant in the tube was measured with a digital caliper to obtain the volume of fluid. Larval body length was used as a covariate to control for the potential allometric effects of larval body size on the amount of fluid produced.

After all measurements, the larvae were reared until pupation. Sex was determined based on pupa size (and verified in adult stage for those that eclosed) following the methods described in Lindstedt et al. (2018). Sex ratios in pine sawflies are strongly female-biased (Craig and Mopper 1993) and we had only a small number of males (*N* = 7) in our sample. Therefore, only the phenotypic data from females were included in the final analysis. Thus, the final dataset consisted of 51 female larvae from 12 families (3 to 5 individuals per family), because one of the initial families was produced by unmated female and included only males decreasing the sample size of larval families from 13 to 12.

### Statistical analyzes

#### The effect of increased conspicuousness and gregariousness on predation risk under natural predator community

A generalized linear mixed model (GLMM) was used to compare larval survival between mesh bag treatment (control for other mortality sources than predation, eg infections) and exposed treatments. The survival of larvae was treated as a binomial response variable (survived/died) and treatment (mesh bag/exposed) as the explanatory variable. Transect was included as a random factor. Survival of larvae in the mesh bag treatments were tested with a generalized linear model (GLM) with a binomial response variable (survived/died) and treatments (solitary/group of 10, and yellow paint/green paint) as explanatory variables.

To test whether the group- and color treatments, or their interactive effects, explained the larval survival (ie individual's likelihood to get killed [1] or survive [0]) in the exposed treatments, we used a Cox proportional hazards model with transect included as a random factor. Since we only had one data point per experimental tree from solitary treatments, it was not possible to include tree ID as a random factor. We excluded the nonsignificant interaction between color and group treatment from the final model (see Table S1 for full-model). Although missing larvae were always replaced the next day to maintain constant proportional hazards, only the first attacks on each experimental branch in the transects were used in the Cox proportional hazards model. This is because the exposure and survival time for added larvae differs from the original ones. However, we also conducted a GLMM with the entire dataset, including replacements, and the results were qualitatively the same as those using the initial attack data (see Table S2). In all models, survival was measured on individual level (eg for a group of 10 larvae, the number of dead larvae was counted, not how many groups were attacked).

Since the sample size in the predation experiment is biased toward group treatments (403 data points, ie individuals) over solitary treatments (40 data points, ie individuals), it can decrease the power of statistical analyses to detect interactive effect of the group size and color treatment in the initial model. Therefore, we analyzed the effect of color on survival also separately within solitary and group size treatment. To do that, we ran two additional Cox proportional hazards models, where we tested the effect of color for the survival within solitary treatment and within group treatment. In both of these models, the transect was again included as a random factor.

#### Phenotypic correlation between *N. sertifer* larval coloration and chemical defense

We used a GLMM to test whether defensive behavior was associated with the coloration (JND values for color contrast and luminance contrast) of the larvae. Larval family was included as a random factor in all models except *U-posture ∼ dorsal color* which were modeled as GLM because of a convergence error with the GLMM model. All coloration values were centered. The defensive behavior variables were binomial: U-posture (yes/no) and deployment of defensive fluid (yes/no). The distribution of the volume of defensive fluid was biased toward small values and contained many zeros. Because data for the volume of defensive fluid was positively skewed and not normally distributed, we modeled the data as gamma distributed with a log link to test whether color and luminance contrasts explained the volume of produced defensive fluid. Length of larva was included in these models to consider allometry. Because gamma distributions do not allow zero values, we set values of 0.001 to replace zeros for individuals that did not produce fluid at all.

In all conducted models, *P* < 0.05 was considered statistically significant. All statistical analyzes were conducted in R version 4.3.2 (R Core Team 2021). Cox proportional hazards models were conducted with package “coxme” with the *coxme(Surv())* function (Therneau 2024) and GLMMs were conducted with package “lme4' with the *glmer* function (Bates et al. 2015).

## Results

### The effect of increased conspicuousness and gregariousness on predation risk under natural predator community

Variation in survival among exposed treatments was caused by predation, as larvae in treatments exposed to predators had significantly lower survival in comparison to larvae protected with the mesh bag (see Table S3). Also, group size or coloration had no effect on larval survival within mesh bags (Table S4).

**Figure 2:**
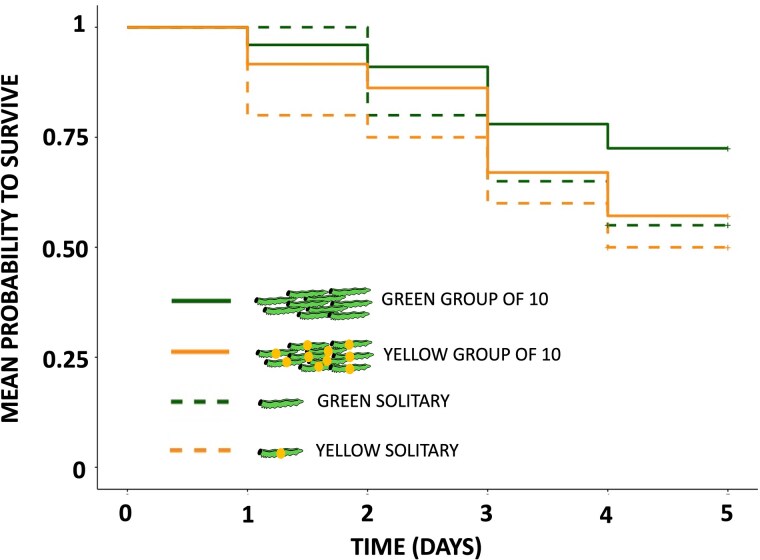
Effect of color treatment (individuals with the natural coloration with the added green spot and individuals with the increased conspicuousness with the added yellow spot) and group treatment (solitary and group) on mean survival against predators during five consecutive days in the field.

In exposed treatments, survival was lower for larvae with the increased conspicuousness of coloration (yellow spot) than for the larvae with natural and more cryptic coloration (green spot) (Table 1, Fig. 2). Group size had no significant effect on larval survival (Table 1, Fig. 2), ie a single larva had an equal chance to survive if solitary or as part of group of 10 larvae. When the effect of color manipulation was tested separately within solitary and group treatments, survival was lower for larvae with the yellow spot than for the control larvae with the green spot in group treatment, but there was no effect of coloration on survival in solitary treatments (Table 1, Fig. 2). Predators never consumed the whole group of larvae (yellow or green painted) during a single day.

**Table 1:** Cox proportional hazard models explaining survival of larvae in the predation experiment.

	Coefficient	Hazard ratio	Std. error	*z* value	*P* value
***Larval survival***					
Group (10 individuals)	−0.429	0.651	0.244	−1.76	0.079
Color (yellow)	**0**.**494**	**1**.**640**	**0**.**161**	**3**.**07**	**0**.**002**
***Larval survival in groups***					
Color (yellow)	**0**.**0537**	**1**.**711**	**0**.**172**	**3**.**11**	**0**.**002**
***Larval survival when solitary***					
Color (yellow)	0.211	1.235	0.460	0.46	0.647

Larval survival is the main model testing the effects of coloration and group size on survival (*n* = 443). Larval survival in groups tests the effect of coloration on mortality of larvae in groups of 10 (*n* = 403). Larval survival when solitary tests the effect of coloration on mortality of solitary larvae (*n* = 40). In Larval survival model, solitary treatment is set as reference category for group size, and in all models, green coloration is set as reference category for coloration. Significant values (*P* < 0.05) are shown in bold.

### Conspicuousness of *N. sertifer* larvae to avian predators (blue tits)

According to the avian vision models, both the ventral and dorsal sides of the larvae should be clearly distinguishable to blue tits even in poor lighting conditions (JND > 3) (Fig. S1). Similarly, birds should be able to distinguish the defense fluid against both dorsal- and ventral sides of the larvae (see Table S5), and against pine needles (color contrast JND = 8.7, luminance contrast JND = 16.4).

### Phenotypic correlation between *N. sertifer* larval coloration and chemical defense

**Figure 3:**
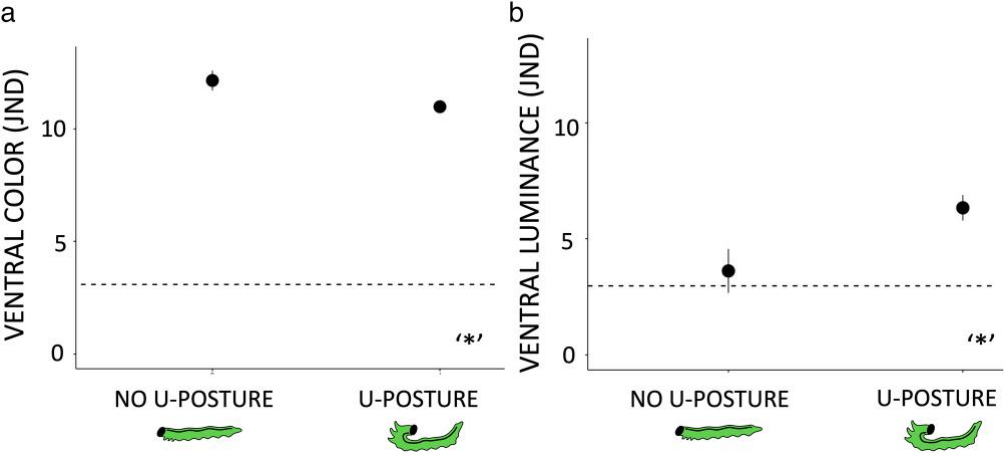
Ventral color contrast a) and ventral luminance contrast b) of *N. sertifer* larvae against the needles of Scots pine (*P. sylvestris*) in individuals that are more likely to display the U-posture and those not displaying the U-posture when provoked. For reference, dashed lines show the threshold value for JND = 3 above which objects should appear clearly conspicuous for blue tits (*C. caeruleus*) (Siddiqi et al. 2004). Significance levels of difference in coloration traits between larvae displaying and not displaying the U-posture are showed with asterisks. Sample sizes of defending larvae *n* = 40 and nondefending larvae *n* = 11. Error bars show means with standard errors.

Larvae that were more likely to perform the U-posture under attack were less conspicuous in terms of dorsal (see Table S6) and ventral color contrast JND values in comparison to individuals that did not do the U-posture under attack (Table 2, Fig. 3). However, larvae that did the U-posture under attack had significantly higher ventral luminance contrast JND values in comparison to larvae that did not do the U-posture (Table 2, Fig. 3). None of the tested color variables differed between the larvae that produced or did not produce the defensive fluid under attack (Table 2, see Table S6 for dorsal coloration) nor did coloration correlate with the volume of produced defense fluid (see Table S7).

**Table 2:** GLMM statistics of models explaining U-posture and deployment of defensive fluid of larvae.

	Estimate	Std. error	*z* value	*P* value
**U-posture**				
*** Ventral color***				
Intercept	**1**.**638**	**0**.**602**	**2**.**718**	**0**.**007**
Color contrast	**−0**.**433**	**0**.**220**	**−1**.**971**	**0**.**049**
*** Ventral luminance***				
Intercept	**1**.**748**	**0**.**644**	**2**.**712**	**0**.**007**
Luminance contrast	**0**.**289**	**0**.**144**	**2**.**006**	**0**.**0448**
**Deployment of defensive fluid**				
*** Ventral color***				
Intercept	1.162	0.855	1.359	0.174
Color contrast	−0.179	0.221	−0.810	0.418
*** Ventral luminance***				
Intercept	1.227	0.946	1.298	0.194
Luminance contrast	0.248	0.152	1.634	0.102

Coloration variables are centered. Significant values (*P* < 0.05) are shown in bold

## Discussion

In this study, we investigated conditions where the evolution of weekly conspicuous coloration combined with chemical defenses could be promoted. First, we show that increased conspicuousness to predators is costly and decreases larval survival by predation in a natural predator community. Second, we found that *N. sertifer* larvae with a uniformly colored ventral side that is revealed during the defensive U-posture are clearly detectable for birds against needle background. Interestingly, our results also suggest that the conspicuousness of ventral side in terms of luminance is associated with the defensive behavior, as individuals more likely to display the U-posture had a brighter (higher luminance contrast) ventral side compared with those that were less likely to adopt the U-posture. On the other hand, these same individuals had on average lower color contrast values. We found no significant association between chemical defense (volume produced or likelihood to deploy the fluid) and ventral (or dorsal) coloration, suggesting that larval coloration does not correlate with the potency of chemical defense.

The costs of being conspicuous were more pronounced in group living than in solitary individuals when the effect of coloration was analyzed separately within group and solitary treatment groups. This suggests that for solitary individuals, the increased conspicuousness does not impact significantly their risk of attack by predators. However, for group living individuals, the increased conspicuousness was more costly suggesting that the combination of gregariousness and conspicuous coloration increases predation risk (Fig. 2). This interactive effect of group and coloration are in accordance with the lack of strong statistical support for the benefits of being in a group irrespective of color (Table 1, see also Lindstedt et al. 2006, 2011).

One reason for the decreased survival in more conspicuously colored groups is that the yellow marking may have increased the conspicuousness of pine sawfly larvae to inexperienced predators (Lindström et al. 2001) and/or to predators that can tolerate or handle the chemical defenses well (Yosef and Whitman 1992). Our experiment was timed to the beginning of the summer (June) matching with the natural occurrence of *N. sertifer* larvae in Central Finland. However, during that time predation pressure toward insect prey can be relatively high as it overlaps with the peak of the breeding season of insectivorous birds (parents need to feed their offspring) and there are already lots of naïve young bird predators around (eg chicks start to leave their nests) (Mappes et al. 2014). As a result, detectability risk for the conspicuous prey can be high (Mappes et al. 2014) and promote weakly conspicuous signal strategies (Endler and Mappes 2004; Medina et al. 2025). Furthermore, while we excluded most of the ant predation to focus more on the visually hunting predators, the diversity of predators is still likely to be high including eg many arthropod predators such as spiders and predatory beetles that are known to vary in their sensitivity to chemical defenses (Burdfield-Steel et al. 2020).

Secondly, the small difference in survival between solitary and group treatment could be partly due to higher variation in predation risk in solitary treatment, as the sample size was ten-times lower in the solitary treatment than in the group treatments. Additionally, detectability risk of solitary larvae is likely to be more stochastic in comparison to group living larvae and gives a realistic view on how predation risks in nature vary. Furthermore, in our experiment we evaluated the survival of solitary larvae and larvae in groups of 10 individuals, which presents the smallest group sizes found from the natural populations. In nature, the larval group size of *N. sertifer* varies from 10 to 60 larvae and can sometimes exceed over 100. Thus, the benefits of group living in terms of diluted predation risk may only arise in larger aggregations than the 10 individuals group used here due to accumulating toxin load or simply via saturation of hunger. This is especially the case if the predator finds larvae unpalatable and stops the attack after consuming only few larvae (Hunter 2000; Ruxton and Sherratt 2006). In future, it would be interesting to test if predation risk of pine sawfly larvae is dependent on the density-stage of the outbreak. For example, terpenes are not necessarily expected to have a very harmful effect in low doses, but their impact is expected to increase in higher doses (Lindstedt et al. 2011, 2022; Price et al. 2011). Thus, if terpene accumulation to predators has a negative impact on their willingness to prey on pine sawfly larvae, we would expect the predation risk to be higher during the low-density stages in comparison to the high-density stages during the pine sawfly outbreak.

Thirdly, it has been suggested that an individual's contribution to responsive defense can be dependent on the group size in a density-dependent manner and explains why researchers often fail in finding the benefit of being in a group in empirical studies (Daly et al. 2012). If contribution to responsive defense is costly for an individual, it might be adaptive to decrease the contribution to chemical defense and rely on the dilution of attack risk in a group instead (Daly et al. 2012). As a result, lower contribution to chemical defense in bigger groups may even out the survival differences between solitary and group treatments also in our experiment. Unfortunately, we did not quantify the differences in defensive behavior of larvae between the group and solitary treatments. However, chemical defense has shown to be costly for pine sawfly larvae (Björkman and Larsson 1991; Lindstedt et al. 2018) and *N. sertifer* larvae have been shown to contribute less for the chemical defense in larger groups (Van Meyel et al. 2025). In addition, contribution to chemical defense increases survival likelihood relatively more in smaller group sizes than in larger group sizes suggesting that investment to collective chemical defense in groups is likely to be density-dependent also in pine sawflies (Van Meyel et al. 2025). Accordingly, it is possible that in our experiment here, the solitary individuals were contributing more to the chemical defense, in comparison to group living individuals which may have diminished the differences between solitary and group treatments.

Our results indicate that one way to balance detectability cost and signaling could be to rely on partly hidden signals (see also Rojas 2017; Umbers et al. 2017; Loeffler-Henry et al. 2023). When threatened, *N. sertifer* larvae expose their ventral side by lifting their head and tail (U-posture) simultaneously while producing defense fluid (Codella and Raffa 1995; Lindstedt et al. 2006, 2011). U-posture is a first stage of the defensive repertoire and a prerequisite for larvae to be able to deploy the defensive fluid. Therefore, individuals taking a U-posture are also alerted and “ready” to move for the next line of defense to deploy the defensive fluid and tap it on the approaching predator. This can be the more critical part of the defensive display: if the risk of being attacked for an individual per se is low, then it may make sense not to deploy the fluid too early and risk to lose it (Lindstedt et al. 2025). Thus, while a bright ventral side may primarily have a countershading function (Donohue et al. 2020), it can additionally function as a hidden signal that is revealed during the U-posture when a bird predator has already detected the larvae and is about to attack. However, color or luminance contrasts did not explain the amount of produced defensive fluid. It is also important to note that we did not measure the quality of defensive fluid in this experiment and thus have no information if the terpene concentration of deployed fluid correlates with the larval coloration. In general, multiple factors can influence on the deterrence and/or terpene concentration of defensive fluid, including the amount of terpenes in the host plant (Larsson et al. 1986; Björkman and Larsson 1991; Codella and Raffa 1995; Lindstedt et al. 2006), or how many times the pine sawfly larva has already deployed and lost the fluid as repetitive production of fluid decreases its concentration (Lindstedt et al. 2018). Smaller volume of fluid can also be more concentrated in mono- and other terpene compounds in comparison to higher volumes (Lindstedt et al. 2018).

It is worth bearing in mind that the avian vision model that was used to determine conspicuousness of coloration to avian predators does not consider eg distance between prey and predator but only mathematically describes how measured color components may affect prey's conspicuousness to a predator (Vorobyev and Osorio 1998; Vorobyev et al. 1998; Troscianko and Stevens 2015). In almost all cases (ventral- and dorsal color contrast and luminance contrast), all the measured values were over the threshold of JND > 3 which stands for likely distinguishable objects. Thus, the avian vision model describes how the mean reflectance of the studied object is viewed by the perceiver and does not give information about the color patterns that may play an important role in forming prey's camouflage (Schaefer and Stobbe 2006; Stevens et al. 2006). Similarly, the avian vision model does not take into account the potential distant-dependent functions of defensive color patterns (ie individuals may appear cryptic from a distance but conspicuous when approaching them close; Tullberg et al. 2005; Bohlin et al. 2008; Loeffler-Henry et al. 2023). Previous studies with different pine sawfly species have shown that predators learn to avoid more efficiently the traditional yellow-black and white-black colored aposematic species (*N. lecontei*) in comparison to green-black colored species (*Diprion pini*) (Lindstedt et al. 2022). Thus, despite appearing clearly detectable for birds, the green-black/grey coloration of *N. sertifer* can still be expected to be less efficient as a warning signal in terms of avoidance learning.

Our results also suggest that the defense fluid is highly discriminable from the ventral side of the larvae and against pine needles, increasing larval conspicuousness to bird predators close by. Selection by predators can sometimes promote only the distinctiveness of unpalatable prey compared with palatable ones, rather than conspicuousness (Merilaita and Ruxton 2007). Considering this, it is possible that displaying the fluid is already a sufficient signal to predators (Skelhorn and Rowe 2006). External defensive compounds can also enable the predator to estimate the palatability of prey based on olfactory and gustatory cues from the defensive compounds without harming the prey (Wiklund and Järvi 1982; Daly et al. 2012; Speed et al. 2012). For example, some earlier literature has reported the collective defensive display (Prop 1960) or volatiles of defensive fluid (Eisner et al. 1974) to be enough to deter predators. Defense fluid also increases the distinctiveness of larvae when encountering a predator, but does not alter conspicuousness permanently. Thus, larvae may rely on their cryptic coloration as the primary defense but when detected by a predator, use the U-posture and defensive fluid as a secondary defense to deter the predator from a close range.

Interactions between predator and prey represent only one of a multitude of interactions that exist within ecological communities. Selective forces outside the predator–prey interaction, such as parasites, pathogens and competitors for food and mates, may affect coloration and chemical defense both directly and/or indirectly. For example, *N. sertifer* larvae have melanistic patterns that may play an important role in thermoregulation (Lindstedt et al. 2009; Hegna et al. 2013). As melanisation is also linked to higher immune investment in insects (Wilson et al. 2001; Fedorka et al. 2013), it can lead to possible indirect selection of integument darkness by pathogens. Also, many herbivores exploit the defense chemicals of their host plant and harness them not only for their own defense against invertebrate and vertebrate predators (Eisner et al. 1974; Codella and Raffa 1995; Speed et al. 2012) but also against parasites and pathogens (Bernays and Singer 2005; Castella et al. 2008; de Roode and Groot 2025). These other functions of defensive chemicals could explain why costly chemical defenses sometimes evolve in species that are already well protected by camouflage. Similarly, group living has not necessarily evolved primarily due to its defense against predators in herbivorous insects, but it can facilitate feeding on host plants and in handling the defensive compounds of hosts (Brian and Faeth 1997; Reader and Hochuli 2003; Fordyce and Nice 2004; Fiorentino et al. 2014). Group living in insect larvae can also play a role in thermoregulation (Qian et al. 2024) enabling individuals in aggregations to maintain stable body temperatures (Klok and Chown 1999). Thus, gregariousness, as well as coloration and chemical defense, are species' characteristics that may have evolved and be maintained by multiple selection pressures operating simultaneously, and the proportional importance of each selection component may vary spatiotemporally. Future studies could aim to disentangle these different selection pressures on coloration and group living, shedding light on conditions where these different selection pressures are operating.

Our results highlight the importance of diverse predator communities in shaping the population dynamics of a forest pest insect. While previous studies have mostly focused on the signal extremes (crypsis and aposematisms), weak- or hidden visual signals are likely common in nature and can play an important role in evolutionary transitions from crypsis to aposematism (Endler and Mappes 2004; Loeffler-Henry et al. 2023). Seemingly cryptic *N. sertifer* larvae seem to have multiple defense mechanisms to avoid predation from the diverse predator community. The diversity of antipredatory strategies in nature creates a good opportunity to study the spatial and temporal conditions in which different selection pressures prevail.

## Supplementary Material

arag010_Supplementary_Data

## Data Availability

Analyses reported in this article can be reproduced using the data provided by Koskenpato et al. (2026).
