# Supplementary material for: High detectability costs select weak warning signals in chemically defended gregarious prey
Source: Behav Ecol. 2026 Feb 2;37(2):arag010. doi: 10.1093/beheco/arag010 (PMC13017696; doi:10.1093/beheco/arag010)
Supplement: arag010_Supplementary_Data [file arag010_supplementary_data.docx]

SUPPLEMENTARY MATERIAL FOR:

**High detectability costs select weak warning signal strategy in chemically defended gregarious prey**

**Effect of increased conspicuousness on predation risk in nature**

***Effect of paint treatment on larval mortality***

Larvae (n = 90) from P-generation were randomly picked from different families and divided into nine groups of ten larvae. These groups were further divided into three different treatments: 1) larvae with a yellow paint mark on the back, 2) larvae with a green paint mark on the back and 3) larva without a paint mark (control), so that there were three larval groups of ten individuals in each treatment. All treatment groups were reared under laboratory conditions with similar diets for seven days and larval mortality was checked daily. If the paint mark was lost e.g. after moulting, it was re-painted. We conducted a generalized linear model (GLM) where survival of larvae was set as binomial response variable (survived / died) and color as categorical explanatory variable (no paint / green / yellow). Mortality of larvae did not differ between painting treatments (χ^2^ = 0.12, df = 2, P = 0.94) and were 8/30, 7/30 and 8/30 dead individuals for non-painted, green painted and yellow painted treatments, respectively.

***Weights of larvae***

We conducted a linear model where larval weight was set as response variable and treatment (yellow painted / green painted / in mesh bags) as categorical explanatory variable. Test was done separately for larvae in groups and solitary larvae, and F-test was calculated for both. Weights differed neither in solitary (F_2,72_ = 0.58, P = 0.56) nor in group (F_2,73_ = 0.10, P = 0.91) treatments.

Table S1. Cox proportional hazard model with interaction group size x color explaining survival of larvae (n=443 [40 solitary and 403 group of ten]) in the predation experiment. Solitary treatment is set as reference category for group size and green coloration is set as reference category for coloration.

|  | Coefficient | Hazard ratio | Std. Error | z value | p value |
| --- | --- | --- | --- | --- | --- |
| Group (10 individuals) | -0.614 | 0.542 | 0.360 | -1.71 | 0.088 |
| Color (yellow) | 0.209 | 1.233 | 0.460 | 0.46 | 0.649 |
| Group size (10) * Color (yellow) | 0.325 | 1.384 | 0.491 | 0.66 | 0.508 |

Table S2. GLMM statistics of a model explaining survival of larvae in the predation experiment with replaced larvae (n=548 [51 solitary and 497 group of ten]) included. Solitary treatment is set as reference category for group size and green coloration is set as reference category for coloration. Significant values (p < 0.05) are shown in bold.

|  | Estimate | Std. Error | z value | p value |
| --- | --- | --- | --- | --- |
| Intercept | **0.701** | **0.311** | **2.251** | **0.024** |
| Group (10 individuals) | 0.480 | 0.306 | 1.569 | 0.117 |
| Color (yellow) | **-0.480** | **0.191** | **-2.515** | **0.012** |

Table S3. GLMM statistics of a model explaining survival of larvae in exposed (experimental) treatments and mesh bag treatments (n=653 [443 exposed and 210 mesh bag]). Exposed treatment is set as a reference category. Significant values (p < 0.05) are shown in bold.

|  | Estimate | Std. Error | z value | p value |
| --- | --- | --- | --- | --- |
| Intercept | **-1.524** | **0.352** | **-4.325** | **<0.001** |
| Mesh bag treatment | **2.087** | **0.294** | **7.095** | **<0.001** |

Table S4. GLM statistics of a model explaining survival of larvae in the predation experiment in the mesh bag treatment (n=210 [20 solitary and 190 group of ten; 100 green painted and 210 yellow painted]. Solitary treatment is set as reference category for group size and green coloration is set as reference category for coloration.

|  | Estimate | Std. Error | z value | p value |
| --- | --- | --- | --- | --- |
| Intercept | 1.812 | 2.218 | 0.817 | 0.414 |
| Group (10 individuals) | -0.365 | 1.072 | -0.341 | 0.734 |
| Color (yellow) | 1.083 | 0.609 | 1.779 | 0.075 |


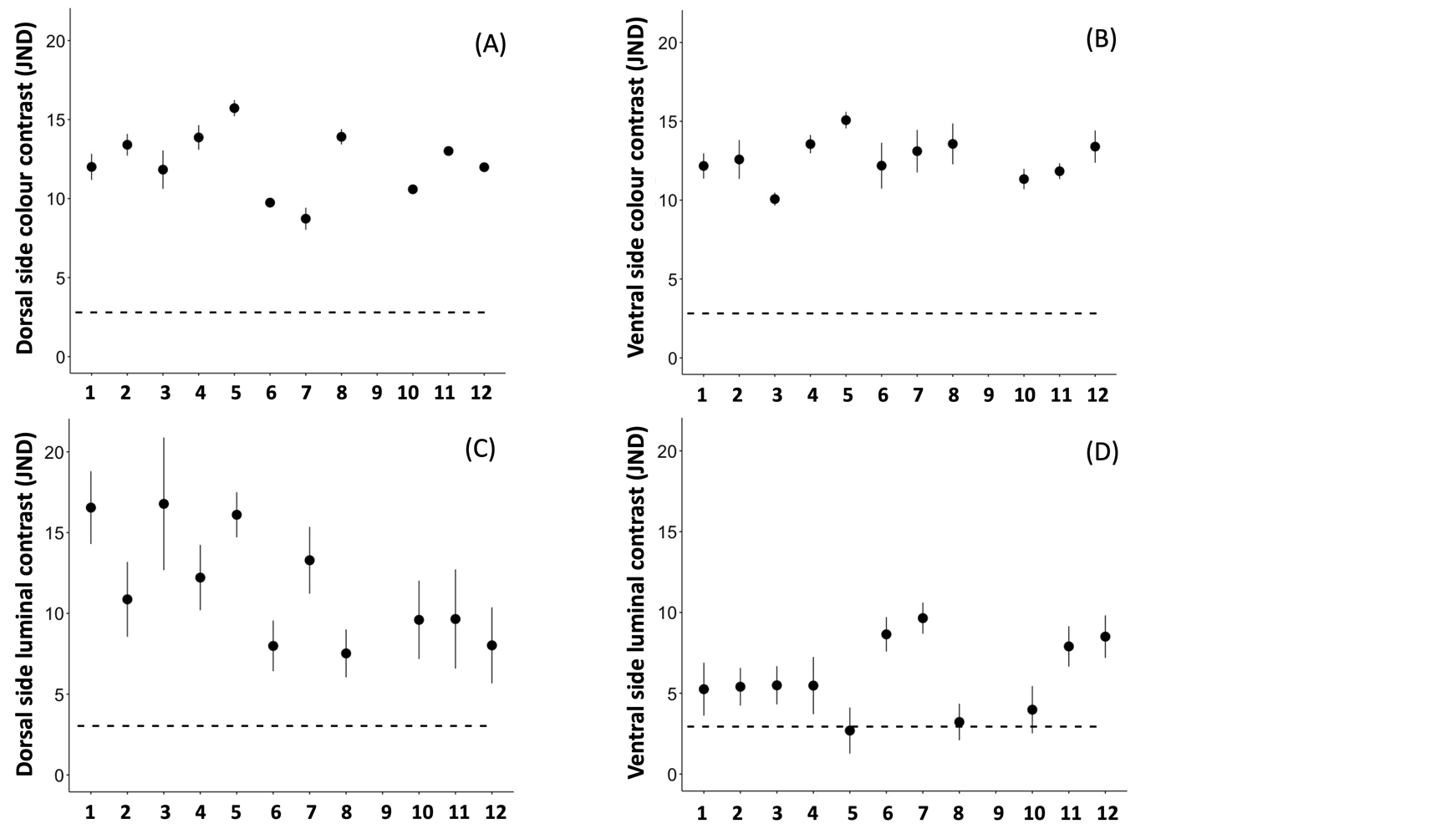


Figure S1. Color contrasts (JNDs) of the dorsal- (12.5 ± 2.2) (A) and ventral (12.8 $\pm$ 2.1) (B) sides of larval family means with standard errors against pine needles. Luminance contrasts (JNDs) of the dorsal- (12.6 $\pm$ 6.3) (C) and ventral (5.8 ± 3.4) (D) sides of larval family means with standard errors against pine needles. Larval families (n=11 [family 9 lacks data]) represented in Y-axes and there were 46 measured larvae altogether. Dashed lines represent the threshold of JND = 3 (values greater than 3 indicate that two objects are likely distinguishable).

Table S5. Color- and luminance contrast values (JNDs) of defensive fluid against family means of dorsal- and ventral side of larvae. Values <1 indicate that the two objects are likely indistinguishable, values 1–3 indicate that the two objects are likely distinguishable but only under optimal light conditions, and values >3 indicate that two objects are likely distinguishable. Values ≥ 3 are bolded.

|  | Fluid against dorsal side JND | | Fluid against ventral side JND | |
| --- | --- | --- | --- | --- |
| Family | Color | Luminance | Color | Luminance |
| 1 | **7.2** | 2.9 | **6.2** | **11.4** |
| 2 | **8.2** | **6.1** | **5.9** | **21.9** |
| 3 | 1.6 | 0.1 | 2.0 | **20.4** |
| 4 | **8.7** | 2.4 | 7.7 | **14.9** |
| 5 | **10.8** | 0.5 | **9.7** | **14.3** |
| 6 | **3.0** | **9.7** | **6.1** | **24.0** |
| 7 | 1.5 | **3.2** | **6.1** | **25.0** |
| 8 | **8.3** | **8.7** | **7.9** | **18.0** |
| 9 | **9.7** | 2.7 | **8.3** | **13.2** |
| 10 | **4.0** | **7.4** | **4.0** | **16.9** |
| 11 | **6.4** | **9.3** | **3.8** | **22.8** |
| 12 | **6.1** | **9.7** | **5.0** | **23.3** |
| 13 | **5.7** | **8.9** | **6.8** | **25.1** |

Table S6. GLMM statistics (GLM for *Dorsal color*) of models explaining U-posture and deployment of defensive fluid of larvae. Coloration variables are centered. Significant values (p < 0.05) are shown in bold.

| **U-posture** | Estimate | | Std. Error | z value | p value |
| --- | --- | --- | --- | --- | --- |
| ***Dorsal colour*** |  |  | |  |  |
| Intercept | **1.470** | **0.415** | | **3.535** | **<0.001** |
| Color contrast | **-0.384** | **0.188** | | **-2.037** | **0.042** |
| ***Dorsal luminance*** |  |  | |  |  |
| Intercept | 2.270 | 1.232 | | 1.843 | 0.065 |
| Luminance contrast | 0.129 | 0.124 | | 1.039 | 0.299 |
| **Deployment of**  **defensive fluid** | Estimate | | Std. Error | z value | p value |
| ***Dorsal colour*** |  |  | |  |  |
| Intercept | 1.110 | 0.819 | | 1.356 | 0.175 |
| Color contrast | -0.273 | 0.263 | | -1.030 | 0.303 |
| ***Dorsal luminance*** |  |  | |  |  |
| Intercept | 1.579 | 1.289 | | 1.225 | 0.221 |
| Luminance contrast | 0.115 | 0.114 | | 1.017 | 0.309 |

Table S7. GLMM statistics of models explaining the volume of produced defensive fluid. Coloration variables and length of larvae are centered.

|  | Estimate | | Std. Error | z value | p value |
| --- | --- | --- | --- | --- | --- |
| ***Dorsal color*** |  |  | |  |  |
| Intercept | **-1.155** | **0.584** | | **-1.977** | **0.048** |
| Larval length | -0.577 | 2.705 | | -0.213 | 0.831 |
| Color contrast | -0.056 | 0.193 | | -0.287 | 0.774 |
| ***Ventral color*** |  |  | |  |  |
| Intercept | -1.162 | 0.602 | | -1.931 | 0.054 |
| Larval length | -1.587 | 2.845 | | -0.558 | 0.577 |
| Color contrast | 0.090 | 0.138 | | 0.654 | 0.513 |
| ***Dorsal luminance*** |  |  | |  |  |
| Intercept | -1.171 | 0.621 | | -1.885 | 0.060 |
| Larval length | -0.491 | 2.659 | | -0.184 | 0.854 |
| Luminal contrast | 0.036 | 0.053 | | 0.674 | 0.500 |
| ***Ventral luminance*** |  |  | |  |  |
| Intercept | **-1.210** | **0.567** | | **-2.135** | **0.033** |
| Larval length | 0.519 | 2.582 | | 0.201 | 0.841 |
| Luminal contrast | 0.149 | 0.102 | | 1.466 | 0.143 |
